# Comparison of physical activity and physical fitness in children and adolescents of Chinese Han and Tibet ethnicity

**DOI:** 10.3389/fpubh.2024.1392803

**Published:** 2024-05-09

**Authors:** Xiaodi Liu, Jiaxing Tang, Weixuan Long, Yu Zou, Jingwang Tan

**Affiliations:** ^1^School of Physical Science and Technology, Ningbo University, Ningbo, China; ^2^School of Physical Education, Shanghai University of Sport, Shanghai, China; ^3^Department of Sports Science, College of Education, Zhejiang University, Hangzhou, China

**Keywords:** physical activity, physical fitness, Tibet ethnicity, Han ethnicity, cross-sectional study, comparative study

## Abstract

**Background:**

Physical activity (PA) and physical fitness (PF) are important markers of health status in children and adolescents in different ethnicities. In this study, we aimed to compare the PA and PF indicators between Tibetan and Han children and adolescents.

**Methods:**

Children and adolescents of 4–9 grades were recruited in Shigatse (*n* = 963) and Shanghai (*n* = 2,525) respectively. The information related to demographic, PA, and PF was collected via a self-reported questionnaire. PA was assessed through the participation of moderate to vigorous PA (MVPA), muscle-strengthening exercise (MSE) and organized sport participation (OSP). PF was estimated using the International Fitness Scale containing components of overall fitness, cardiorespiratory fitness, speed and agility, muscular strength and flexibility.

**Results:**

Han (mean age = 13.45 ± 3.3 years; 49.7% girls) and Tibet (mean age = 13.8 ± 2.5 years; 48.3% girls) children and adolescents from Shanghai and Shigatse completed the questionnaire survey. It was revealed that Tibetan students had higher MVPA, MSE and OSP than children and adolescents of Han ethnicity (*p* < 0.01, small to medium effect size). A relatively higher percentage of student in Shanghai did not participate in any form of PA. On the other hand, less Tibetan students thought their PF indicators including overall fitness, cardiorespiratory fitness, speed and agility, muscular strength and flexibility were poor or very poor than their counterparts of Han ethnicity (*p* < 0.01, small to medium effect size).

**Conclusion:**

Tibetan children and adolescents have higher levels of PA and PF in comparison to their Han counterparts. More children and adolescents of Han ethnicity engage in no PA and think their PF indicators were poor.

## Background

Physical activity (PA) was defined as any bodily movement produced by skeletal muscles that requires energy expenditure (1). It has been demonstrated that PA was closely related to the health outcomes of children and adolescents (2–4). For instance, the engagement of PA was associated with the reduced incidence of obesity (5), improvement of the mental health (6) among children and adolescents and even was beneficial to decreasing cardiovascular risk in their adulthood (7). Despite these benefits, a very high prevalence of physical inactivity (PI) still remains. It has been confirmed that the majority of adolescents do not meet current physical activity guidelines (8). As a result, the insufficiency of PA could be treated as a serious threat to the health and wellbeing of children and adolescents. Physical fitness (PF) is the capacity to perform PA, and it refers to a full range of physiological and psychological qualities (9). PF can be divided into health-related and skill-related components. Health-related PF primarily focuses on health outcomes, while skill-related PF is associated with motor skill performance such as speed, power, coordination, reaction time, agility and balance (10). PF can be seen as a useful health marker during childhood and adolescence (9, 11, 12). Additionally, PF is also closely correlated to academic performance (4), depression (13) and health-related quality of life of children and adolescents (14). Despite the importance of PA and PF, lower levels of PA and PF have been evidently documented among Chinese children and adolescents (15, 16). Only around one third (29.9%) of Chinese children and adolescents met the well-recognized PA guideline, which was a minimum of 60 min of moderate to vigorous physical activity (MVPA) per day (17, 18). With respect to PF, only 3 in 10 Chinese school-aged children achieved an “excellent” or “good” fitness mark set by national physical fitness standards (19). In response to these health issues, *Healthy China 2030* blueprint was conducted in which the goals was to let Chinese school-aged children achieve at least 1 h of PA per day and having more than 25% of them achieve an “excellent” rating in fitness (15, 20).

Ethnicity is an important factor affecting PA engagement among children and adolescents. Previous studies found that minority subpopulations of US adolescents had lower PA level compared with their peers of other ethnicities in the USA, and the absence of PA was correlated with the increased overweight prevalence (21, 22). Similarly, a 5-year longitudinal study performed in London also showed that Asian students were less active than whites (23). The ethnical differences of PA may be considered as one of contributors to health behaviors inequality between different ethnicities. Further, these differences highlight the necessity of enacting targeted PA policies to improve the overall health for children and adolescents of all ethnicities. China is a multi-ethnic country in which Han ethnicity accounting for the majority (91%) of the whole population. Tibetan people, one of the minorities in China, primarily reside in Tibet autonomous region located on the western part of China and this area is the highest areas on the earth with high-altitude hypoxia environment (24). Shigatse is the second largest city in Tibet autonomous region and around 90% residents in this city are Tibetan people. Although nearly sharing the same latitude, the city of Shanghai of China has different altitude, climate, socio-economic conditions and population composition in contrast to Shigatse (25). The differences between these two areas may not only influence the PA participation of children and adolescents, but also lead to the health inequality. To date, knowledge in relation to the difference of PA and PF between Tibet and Han ethnicity has not been well established. It was previously reported that 9.1% of children and adolescents living in Lhasa (a city in Tibet) accumulated at least 60 min of MVPA daily when measured by accelerometer and Tibetans were more active than Hans there (25). In contrast, questionnaire surveys conducted in Shanghai found that around 20% of those met the standard (26, 27). The difference existed in these studies may due to several reasons such as the tool, sample and cut-point, which leads to the necessity to research this question in a uniform way. Up to now, no study has been done to directly compare the MVPA level and other forms of PA including muscle-strengthening exercise (MSE) and organized sport participation (OSP) between children and adolescents of Tibet and Han ethnicity. As for the PF, it was found that Tibetan children and adolescents had superiority on abdominal strength but not lower limb strength than their Chinese counterparts (28). In comparison, cardiorespiratory fitness of Tibetan children and adolescents was lower compared with their peers living in Shanghai (29). Similar to PA, evidence related to the difference of PF among two ethnicities has not been gathered in a direct way, probably resulting in inaccurate estimations. In addition, evidence concerning the difference of other components of PF (i.e., flexibility, speed and agility) lacks between Tibetan and Han students. To understand the ethnic disparity of PA and PF, we conducted a cross-sectional study through collecting self-report data of children and adolescents from Tibet ethnicity in Shigatse and Han ethnicity in Shanghai.

## Methods

### Study design

This study was a cross-sectional questionnaire survey conducted by using method of random cluster sampling. The procedure of data collection started on October 2 2023, and finished within the last week of November 2023. The sample size was calculated using G*power software package (version 3.1.9, Heinrich-Heine-Universität Düsseldorf, Düsseldorf, Germany) with the parameters including effect size (0.3), α level (0.05), power (0.95), and degree of freedom (4) respectively. The minimum sample size was 870 given by the software. The present study was carried out in line with the Declaration of Helsinki. The Institutional Review Board (or Ethics Committee) of the Shanghai University of Sport approved the protocol of this study (ethical code: 102772021RT071).

### Participants

Children and adolescents from 3 randomly selected primary and middle schools in Shanghai and Shigatse, respectively, participated in this questionnaire survey. Inclusion criteria used in the recruitment process were as follows: (1) grade 4–9; (2) no serious physiological or psychological disorders affecting PA participation over the past 12 months; (3) voluntarily participated in the questionnaire survey with written informed consent approved by parents.

### Procedures

As this study was supported by the local education commission of Tibet and Shanghai, all targeted schools (i.e., primary and middle schools) were randomly selected based on the premise that the survey would not influence schools’ routines. The questionnaire survey was conducted by a professional staff in the targeted school. All children and adolescents were told with the requirements and rights before filling in the questionnaire. Then, each child and adolescent in this study voluntarily completed the questionnaire using the electronic equipment (e.g., computers, mobile phones). The questionnaire consisted of three parts: demographic information, self-reported PA and self-reported PF. For students who were inaccessible to electronic devices, they were encouraged to use the equipment of their parents. If all forms of electronic devices were unfeasible, students were allowed to complete the paper questionnaire which was manually transformed into electronic version by the school staff. All data was collected and analyzed in an anonymous way.

## Measures

### Demographics

As the first part of the questionnaire, demographic information was collected including age, gender, grade, residence, education of parents and family type. In the present study, the age of 13 was treated as a cut-point to distinguish children and adolescents. The family type referred to whether participants lived with their parents (not single father or mother).

### Physical activity

In the current study, the PA of children and adolescents was estimated in three parts (i.e., MVPA, MSE and OSP) with one item in each part. The internal consistency reliability for these items was 0.712 measured with Cronbach’s α coefficient. MVPA was investigated by asking participants “Over the past 7 days, on how many days did you engage in MVPA for at least 60 min per day?” This item had 8 answer choice from 0 to 7 days a week (i.e., 1. none, 2. 1-day, 3. 2-day, 4. 3-day, 5. 4-day, 6. 5-day, 7. 6-day, and 8. 7-day). MVPA was defined as at least moderately breathtaking and sweating in the daily life, physical education class and other organized exercises (30). According to previous studies, the test–retest reliability of this item was satisfactory in Chinese students (31) and the concurrent validity was acceptable (32).

MSE referred to any activities that increase skeletal muscle strength, power, endurance, and mass (e.g., strength training, resistance training, or muscular strength and endurance exercises) (17). This item was asked with the question: “In the past week, how many days did you engage in exercise to strengthen the muscle, such as push-ups, sit-ups, or lifting weights?” The possible responses were: “1. none, 2. 1-day, 3. 2-day, 4. 3-day, 5. 4-day, 6. 5-day, 7. 6-day, and 8. 7-day.” This item has been widely used in several countries and was tested as a reliable and valid tool (33–35).

OSP meant the activities organized by sport clubs, school team or any other cultural or religious events. In this item, students were asked “Whether you had participated in organized sport programs over the past 12 months?.” This question had 4 answer choices: 1. none, 2. 1–3 times a month, 3. 1–2 times a week, 4. at least 3 times a week. This question has been validated among Chinese children and adolescents in previous study (26).

### Physical fitness

In the present study, PF was assessed with International Fitness Scale, Chinese-version (IFIS-C), which has been justified as a reliable instrument for estimating PF in Chinese children and adolescents (36). The Cronbach’s α coefficient was 0.738 for the PF domain. In addition, it was also tested to be valid in existed researches (37, 38). In this questionnaire, five components including overall fitness, cardiorespiratory fitness, speed and agility, muscular strength, and flexibility were asked with 5-point Likert scale (i.e., “very poor,” “poor,” “average,” “good,” and “very good”).

### Statistical analysis

All data was analyzed by using the software of SPSS (version 25, IBM Corporation, Armonk, NY, United States). Descriptive statistics was used to report demographic information of the participants, in which age was given as mean ± standard deviations, and other indicators were given as numbers or percentages. The difference of PA and PF between Han and Tibet children and adolescents was examined by chi-square test. The Cramer’ s V (V) was used to calculate the effect size of the chi square test. V < 0.1, 0.1–0.3, 0.3–0.5, and > 0.5 denoted small, small to medium, medium to large and large effect size (39). In this study, the level of statistical significance was set at *p* < 0.05.

## Results

After deleting missing data, questionnaires from 963 participants in Tibet (48.3% girls) and 2,525 students in Shanghai (49.7% girls) were finally collected and analyzed. All participants in Shigatse and Shanghai were aboriginal Tibetan and Han ethnicity, respectively. As shown in Table 1, the average age of Han participants is 13.45 compared with 13.80 of Tibetan students. The majority of students (89%) in Shanghai are from urban area, whereas 82% of participants come from rural areas in Shigatse. In terms of the education level of parents, 42.9% of parents in Tibet have diploma of junior middle school or under, while this number is relatively low in terms of Han parents (33.6%). Considering the number of parents whose education level are at least high school, the quantity of Han parents (66.4%) is obviously higher in contrast to that of Tibetan students’ parents (57.1%).

**Table 1:** Demographic information of participants.

	Ethnicity	Gender		Age		Residence		Family type*		Education level of parents			
		Boy	Girl	Boy	Girl	Urban	Rural	Yes	No	JMSU	JHS	UB	UG
Ethnicity	Han	1,270 (50.3)	1,255 (49.7)	13.4 (4.2)	13.5 (2.3)	2,247 (89)	278 (11)	2,138 (84.7)	387 (15.3)	849 (33.6)	676 (26.8)	860 (34.1)	140 (5.5)
	Tibet	501 (51.7)	462 (48.3)	13.7 (2.1)	13.9 (2.8)	125 (18)	838 (82)	891 (92.5)	72 (7.5)	413 (42.9)	221 (22.9)	284 (29.5)	45 (4.7)

JMSU, Junior middle school or under; JHS, Junior high school; UB, University bachelor; UG, University graduate. *Whether children or adolescents lived with their parents (not single father or mother); gender, residence, family type and education level of parents were presented as n (%), age was presented as mean value (standard deviation).

**Figure 1:**
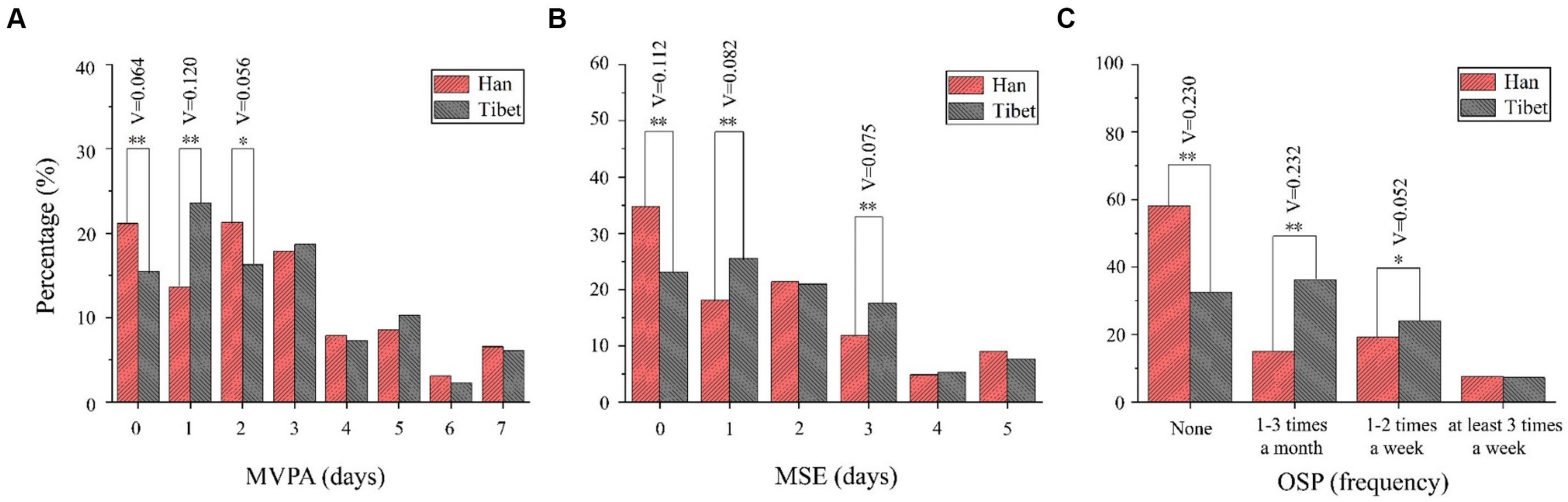
Comparison of **(A)** MVPA, **(B)** MSE, and **(C)** OSP between children and adolescents of Han and Tibet ethnicity. *p < 0.05; **p < 0.01. V: Cramer’ s V. MVPA, moderate to vigorous physical activity; MSE, muscle-strengthening exercise; OSP, organized sport participation.

The days of engaging in MVPA, MSE, and OSP across the week are presented in Figure 1. There is significant difference of MVPA between children and adolescents of these two ethnicities (Figure 1A). To be specific, the number of Han students having 0 day of MVPA was higher (21.15%) than their counterparts of Tibet ethnicity (14.45%, V = 0.064), while the opposite difference is found as for the choice of 1-day (V = 0.120) a week. Students of these two ethnicities possess similar percentage on choices of at least 3-day MVPA per week. The difference of MSE between students of the two ethnicities is similar to the MVPA (Figure 1B). There are more Han students (34.73%) who did not participate any MSE across the week in comparison to Tibetan students (23.09%) (V = 0.112). On the other hand, children and adolescents in Tibet have generally high frequency of participating MSE at least 1-day a week (V = 0.082) and 3-day a week (V = 0.075). In Figure 1C, the percentage of students having no OSP of Han ethnicity nearly doubles compared with that of Tibetan students (58.18% versus 32.5%, V = 0.230). In contrast, revealed by the frequency of 1–3 times per month (V = 0.232) and 1–2 times per week (V = 0.052), Tibetan children and adolescents have more OSP than those of Han ethnicity.

Table 2 presents the results with regard to components of PF between ethnicity of Han and Tibet. It is shown that the difference of each component is significant (*p* < 0.01). As for the overall fitness, the percentage of Han students reporting “poor” and “very poor” is higher compared with that of Han ethnicity (V = 0.127), which is in line with the outcome of cardiorespiratory fitness (V = 0.136). Although nearly the equal proportion of children and adolescents (30% in two ethnicities) considered their muscle strength is “good” or “very good,” a larger percentage of participants of Han ethnicity believed that they had poor muscle strength (V = 0.126). With respect to the item of speed and agility, Tibetan participants have higher percentage for the marker of “average,” “good” and “very good,” but lower proportion for the “poor” and “very poor” marker (V = 0.087). As fewer Tibetan children and adolescents thought their flexibility are “poor” or “very poor,” the difference of flexibility has the largest effect size (V = 0.225) between these two ethnicities.

**Table 2:** Comparison of self-rated physical fitness between children and adolescents of Han and Tibet ethnicity.

	Ethnicity	Very poor	Poor	Average	Good	Very good	*P*	V
Overall fitness	Han	70 (2.77)	303 (12.00)	1,324 (52.44)	620 (24.55)	208 (8.24)	<0.01	0.127
	Tibet	12 (1.25)	41 (4.26)	549 (57.02)	323 (33.54)	38 (3.95)		
Cardiovascular health	Han	81 (3.21)	353 (13.98)	1,196 (47.37)	666 (26.38)	229 (9.07)	<0.01	0.136
	Tibet	15 (1.56)	48 (4.98)	534 (55.45)	330 (34.27)	36 (3.74)		
Muscle strength	Han	83 (3.29)	389 (15.41)	1,288 (51.01)	594 (23.52)	171 (6.77)	<0.01	0.126
	Tibet	14 (1.45)	67 (6.96)	592 (61.47)	258 (26.79)	32 (3.32)		
Speed and agility	Han	58 (2.30)	313 (12.40)	1,173 (46.46)	702 (27.80)	279 (11.05)	<0.01	0.087
	Tibet	17 (1.77)	62 (6.44)	535 (55.56)	310 (32.19)	39 (4.05)		
Flexibility	Han	168 (6.65)	554 (21.94)	1,097 (43.45)	506 (20.04)	200 (7.92)	<0.01	0.225
	Tibet	9 (0.93)	63 (6.54)	554 (57.32)	270 (28.04)	67 (6.96)		

Data presented as n (%); V: Cramer’ s V; the calculation of P and V are based on the proportion of Han or Tibet children and adolescents with self-rated physical fitness indicators of “poor” and “very poor.”

## Discussion

The purpose of the current study was to compare the PA and PF of children and adolescents between Han ethnicity living in Shanghai and Tibet ethnicity residing in Shigatse. The results revealed that Tibetan children and adolescents showed higher PA and PF than those of Han ethnicity. Children and adolescents of Han ethnicity were more inactive and have poorer PF, whereas Tibetan children and adolescents have higher PA and better PF.

Tibetan children and adolescents are generally active than their counterparts of Han ethnicity. Based on the data collected by the accelerometer, Tibetan children and adolescents displayed higher PA level than their Han peers living in plain cities (25), which is in line with the results of current study. In contrast, demonstrated by Chen et al. (40), more Han students in Shanghai were inactive. When compared with other regions across the world such as USA 24.3% (41), Australia 23.5% (42) and Malta 24.5% (43), the prevalence of MVPA in both ethnicities are low and far from meet the WHO guideline (25). Similar to MVPA, Tibetan children and adolescents possessed better MSE than their Han counterparts. However, only 30.37% of Tibetan students and 25.74% of students of Han ethnicity meet the recommended volume of MSE (at least 3 days per week) and the percentages are lower than the one given by a national investigation of China (39.3%) (44). Consistent with Johnston et al. who reported the difference of OSP between ethnicities (45), the high level of OSP among Tibetan children and adolescents was also found in the current study. Actually, the lack of OSP among children and adolescents in Shanghai has been reported in 2016, which may be used to partly interpret the OSP disparity of the present study (26).

There may be several reasons for the differences of PA between children and adolescents of Han and Tibet ethnicity. First, the cultural attributes in Tibet may be potentially helpful to explain the differences. According to the previous study, Tibetan children and adolescents had more opportunity to participate traditional cultural and religious activities such as long kowtow and dance (46, 47). All of these activities could be thought as one of contributors to the increased participation of MVPA, MSE, and OSP among Tibetan school-aged students (48, 49). On the contrary, Han children and adolescents in Shanghai were mainly sedentary and lacked organized OSP (26). The inadequate PA of participants in Shanghai may be explained by several factors including homework load, academic pressure, and the lack of sport clubs and space in the community (26, 40). Second, residential location (e.g., urban or rural area) probably affects the PA participation in both ethnicities. According to previous studies, the disparity of students’ PA participation existed between urban and rural area (18, 50). In the present study, larger number of Tibetan children and adolescents (82%) lived in rural area in contrast to Han students mostly (89%) residing in urban areas, possibly indicating that Tibetan students may have more space and chances to engage in the traditional sport activities and subsequently gained higher PA level. However, it is not clear whether other factors (e.g., sports facilities, transport to school) brought by residential disparity have also significantly affected Tibetan students’ PA participation. Third, it has been demonstrated that parental support such as encouragement, accompanying and role-model from parents was beneficial to facilitating PA participation among children and adolescents (51, 52). In this study, higher percentage of Tibetan students (92.5%) lived with their parents than their peers of Han ethnicity (84.7%), probably meaning that Tibetan students possessed more opportunities of receiving parental encouragement, accompanying and involvement in contrast with their Han counterparts. Fourth, the existed evidence showed that higher level of parents’ education was positively related to the PA participation of adolescents (53). Although the proportion of Han parents with at least graduate diploma was higher than that of Tibetan parents, we did not observe the superiority of PA level of Han students except the outcome of MVPA. Facing this inconsistency, future studies seem warranted to explore the specific reason. Apart from the above social-ecological factor, physical adaptation to high-altitude environment may also played an important role. It has been reported that Tibetan people had higher pulmonary volumes, aerobic capacity and gas exchange efficiency, which could make them tireless in PA participation such as walking and climbing (54).

Similar to the difference of PA between Han and Tibet children and adolescents, Tibetan children and adolescents demonstrated superiority in each component of PF than their counterparts of Han ethnicity. To be specific, more Han students reported that their PF components were “poor,” whereas high proportion of Tibetan participants rated “good” for their PF components. Another cross-sectional study observed that Tibetan children and adolescents had lower cardiorespiratory fitness than those living in Shanghai (29), which was inconsistent with the result of the present study. In this study, 20 m shuttle run test rather than subjective questionnaire was used to measure cardiovascular capacity, which may explain the discrepancy to an extent. Previous study investigating muscle strength of Tibetan people demonstrated that Tibetan children and adolescents have relatively strong abdominal strength, but lower limb strength (28), suggesting that the self-reported muscle strength in the current study may be affected by strength perception on different part of the body. As for the other components including speed, agility and flexibility, the superiority was found in Tibetan children and adolescents in contrast to Han students. In fact, limited evidence has been reported concerning speed, agility and flexibility of Tibetan students. Previous national PF evaluation in China revealed that the majority of Chinese children were at the mark of “pass” (59.9%) rather than “good” (25.8%) and “excellent” (5.95%), but they did not include children and adolescents from Tibet (19). Another nationally representative survey (2005–2014) found that children and adolescents living in the western region of China including Gansu, Qinghai, and Tibet had lower PF (i.e., forced vital capacity, standing long jump, sit-and-reach, body muscle strength, 50-meter dash and endurance running) than those living in the eastern region of China such as Beijing, Zhejiang, and Shanghai (55). However, this study also observed that there has been a decreasing trend in terms of the low-level PF proportion in Chinese western areas, possibly indicating that children and adolescents in Tibet may have gradually increased their PF level over the past several years.

Some factors could be considered to interpret the difference of PF between Han and Tibet ethnicity. It has been proposed that higher level of PA was significantly associated with better PF (56, 57). Hence, factors beneficial to the increase of PA may also be used to explain the difference of PF (19, 58). On the other hand, physiological adaptation in high altitude could also be considered. To maintain the normal levels of oxygen use in the hypoxia environment, indigenous Tibetan residents encompassed higher capillary density and blood flow (59, 60). Additionally, Tibetans can maintain higher cerebral tissue oxygenation during maximal normoxic exercise and enhance muscle oxygen utilization during hypoxic exercise, showing better energy economy at exercise exertion (61–63). The genetic adaptation, such as the EGLN1 and PPARA among indigenously Tibetan students can be regarded as another mechanism for their superior PF components (64, 65). Hence, genetic changes of indigenous Tibetan students may result in physiological adaptation to hypoxia environment and provide better exercise competence.

## Limitation

Some limitations should be mentioned in the present study. First, because of the lower population density in Tibet, students of Tibet ethnicity were not proportionally selected in comparison to Han ethnicity of Shanghai. On the other hand, the PA and PF were assessed with questionnaires instead of objective measures such as accelerometers, which may influence the accuracy of estimation. Finally, no interventional programs have been performed despite the identified disparity between these two ethnicities.

## Conclusion

In summary, Tibetan children and adolescents in Shigatse have higher level of PA and PF level in comparison to their Han counterparts living in Shanghai. Social-ecological factors rather than high-altitude environment may played more important roles in affecting PA and PF of Tibetan children and adolescents. In practice, policies and intervention strategies should focus more on reducing the proportion of children and adolescents who participated none PA weekly and answered poor PF. Future studies are still warranted to investigate the impact of different altitudes on PA and PF.

## Data availability statement

The raw data supporting the conclusions of this article will be made available by the authors, without undue reservation.

## Ethics statement

The studies involving humans were approved by the Institutional Review Board (or Ethics Committee) of the Shanghai University of Sport. The studies were conducted in accordance with the local legislation and institutional requirements. Written informed consent for participation in this study was provided by the participants' legal guardians/next of kin.

## Author contributions

XL: Validation, Supervision, Project administration, Formal analysis, Writing – original draft. JXT: Visualization, Resources, Formal analysis, Writing – original draft. WL: Visualization, Writing – original draft. YZ: Supervision, Conceptualization, Writing – original draft. JWT: Supervision, Resources, Conceptualization, Writing – review & editing.
